# A mixed-methods feasibility study of dog-assisted exercise therapy in geriatric inpatients

**DOI:** 10.1186/s12877-026-08157-4

**Published:** 2026-08-29

**Authors:** Lilly Joschko, Eva Jansen, Stefanie Weinert, Maximilian Hinse, Roland Zerm, Harald Matthes

**Affiliations:** 1 Institute of Social Medicine, Epidemiology and Health Economics, Charité – Universitätsmedizin Berlin, corporate member of Freie Universität Berlin and Humboldt - Universität Zu Berlin, Berlin, Germany; 2 Hospital Gemeinschaftskrankenhaus Havelhöhe, Geriatric Clinic, Berlin, Germany; 3 Research Institute Havelhoehe (FIH), Berlin, Germany; 4 Department of Child and Adolescent Psychiatry and Psychotherapy, Otto-Von-Guericke-University Magdeburg, Magdeburg, Germany

**Keywords:** AAT, RCT, Mobility, Motivation, Satisfaction, Depression

## Abstract

**Background:**

Animal-assisted therapy (AAT) is a promising complementary approach in geriatric rehabilitation, but evidence on feasibility and patient experiences remains limited.

**Methods:**

This exploratory, mixed-methods, two-arm randomized controlled parallel-group design (RCT) compared dog-assisted exercise therapy (intervention) to standard exercise therapy (active control) in geriatric inpatients during a two-week rehabilitation program. Quantitative data on mobility (TUG), depression (HADS-D) symptoms, motivation (VAS), and satisfaction (VAS)were collected and analysed using descriptive statistics, cross-tabulations, group comparisons (unpaired t-test or Mann–Whitney U test), and correlation (Spearman’s ρ). Semi-structured interviews explored patientsexperiences. Data were analyzed separately and integrated using a joint display approach.

**Results:**

27 patients participated in the RCT, and 11 in the embedded qualitative study. Quantitative measures showed no clinically meaningful between-group differences in mobility or depressive symptoms: mobility (TUG) (control: Mdn = 16.00, IQR [14.25, 22.50]; intervention: Mdn = 18.00, IQR [13.00, 20.50]); depression (HADS-D) = 0.2 points (95% CI [–3.2, 3.6], SD = 3.05). Motivation and satisfaction were consistently high across conditions. A strong positive correlation was observed between motivation and satisfaction (ρ = 0.65, *p* < .001). Qualitative results indicated high acceptance and feasibility, enhanced emotional well-being, motivation, social interaction, and engagement, particularly associated with the presence of the therapy dog. Mixed-methods integration indicated high feasibility and acceptability of the intervention. Qualitative findings complemented the quantitative results by demonstrating that the therapy dog enhanced the ward atmosphere and patient engagement, despite the absence of measurable short-term clinical changes.

**Limitations:**

Key limitations include novelty effects, the dog’s visibility across conditions, the influence of prior pet ownership, COVID-19, dropouts, small sample size, the awareness of the presence of the dog at ward for the control group, and a heterogeneity group of conditions. These factors may have impacted the overall outcomes.

**Conclusion:**

Animal-assisted exercise therapy is feasible and well accepted in geriatric inpatient care. Its primary benefits appear to be experiential and psychosocial, supporting engagement and rehabilitation participation rather than immediate clinical improvement. Further research should investigate long-term effects and underlying mechanisms.

**Trial registration:**

This study has been registered in the German Clinical Trial Registry with trial ID DRKS00024955 on 22 April 2021 and was approved by the ethics committee (EA4/071/21).

## Introduction

Animal-assisted therapy (AAT) has emerged as a promising complementary approach in geriatric care, showing positive effects on various aspects of well-being. Previous studies have highlighted benefits such as improved social functioning, reduced depressive symptoms, and loneliness, enhanced communication, and better psychosocial and physiological outcomes [2, 6, 7, 20]. Furthermore, AAT has been found to reduce pain, anxiety, depression, and loneliness, while improving mood and quality of life [12, 35].

Older adults have an increased risk of social isolation and loneliness [26, 31, 38]. Loneliness, recognized as a detrimental factor for the mental health of older adults, may also increase the risk of developing depression [5]. Research suggests that the subjective emotional experience of loneliness had a stronger impact on mental health than objective social isolation, with long-term effects such as persistent depressive symptoms [26, 31]. Older adults showed a prevalence of depression of 5.7% [4.94–6.55%] [15]. Globally, depression is one of the most disabling mental disorders, ranking among the top 25 causes of death worldwide [15]. In contrast, a review and meta-analysis by Jain et al. [20] and Villarreal-Zegarra et al. [43] found that AAT involving dogs significantly improved depressive symptoms and reduced feelings of loneliness.

AAT has been increasingly examined as a complementary approach to enhance mobility and functional independence in older adults. The review by Krause-Parello and colleagues suggests that engagement with animals is associated with improvements in cardiovascular parameters and greater levels of physical activity, both of which are critical determinants of physical performance and autonomy [24]. Furthermore, a systematic review by Chang et al. [10] identified beneficial effects of AAT across physiological, psychosocial, cognitive, and behavioral domains in older adults. In a case study in a rehabilitation context, AAT has also been integrated into physical therapy to promote participation, reduce perceived pain, and facilitate attainment of functional goals, thereby supporting mobility and independence [11].

In geriatric inpatient care, the primary goal is to restore patients' independence and mobility, promoting successful aging [32]. Despite the promising evidence for AAT, its integration into standardized exercise therapy for hospitalized geriatric inpatients remains insufficiently investigated, particularly regarding feasibility, and patients’ subjective experiences. Therefore, this study aimed to evaluate the fesability of exercise therapy with or without a therapy dog in geriatric inpatients. Quantitatively, we assessed feasibility-relevant descriptive outcomesof dog-assisted therapy on mobility, motivation, satisfaction, and depression. Qualitatively, we explored participants’ experiences within the exercise therapy, the therapy dog and rehabilitation context, beyond predefined outcome measures. Additionally, potential harms, including adverse physical or psychological reactions, were monitored, with the hypothesis that the intervention would be safe and well tolerated. By integrating quantitative and qualitative data, this mixed-methods approach provides a comprehensive assessment of both clinical outcomes and patient-reported experiences. This study seeks to contribute to the growing body of evidence supporting innovative, and patient-centered approaches to geriatric care, offering insights into AAT.

## Methods

### Study design

We conducted a two-arm randomized parallel-group mixed-medthods study to investigate the feasibility of dog-assisted exercise therapy (AAT; intervention group) versus conventional exercise therapy (active control) in patients undergoing a two-week acute geriatric inpatient rehabilitation program. Quantitative and qualitative data were collected concurrently from the same participants, analyzed separately, and then integrated using established mixed-methods procedures.

This study was designed as a feasibility trial, with the primary aim of evaluating key feasibility parameters, including recruitment and retention rates, intervention delivery and adherence, safety, and participants’ subjective experiences. These outcomes were used to assess the practicality and acceptability of implementing dog-assisted exercise therapy within a routine inpatient rehabilitation and for identifying procedural refinements required for a future definitive trial.

Participants were randomly allocated to the intervention or active control group using a pre-specified randomization procedure to ensure balanced group assignment. The use of randomization in this feasibility context was not intended to evaluate intervention efficacy, but rather to assess the feasibility of implementing a randomized controlled design under real-world clinical conditions, including recruitment into randomized arms, acceptability of group allocation procedures, and feasibility of study logistics (e.g., scheduling, intervention delivery, and data collection across groups). In addition, randomization allows for a structured and unbiased comparison of feasibility parameters between groups, which is important for identifying potential group-related differences in feasibility outcomes (e.g., adherence or dropout patterns) that may inform the design of a subsequent definitive trial.

Although the study was not powered to detect statistically significant between-group differences in clinical outcomes, between-group analyses were conducted in an exploratory and descriptive manner only. The aim of these analyses was not hypothesis testing, but the estimation of outcome variability and the exploration of potential trends in outcome trajectories across groups. These estimates are used to inform sample size calculations, outcome selection, and methodological refinement for a future adequately powered randomized controlled trial.Integration was guided by established mixed-methods procedures for merging data in health research [13, 14]. Specifically, we conducted a structured cross-strand comparison of quantitative outcomes with qualitative themes derived from patient interviews. Integration involved three steps: (1) convergence checks to identify areas of agreement or divergence between numerical outcomes and patient-reported experiences,(2) explanatory comparisons to contextualize quantitative patterns using qualitative insights; and (3) the development of a joint display, which visually aligned objective measures with participants’ perceptions of therapy acceptability and experience across the rehabilitation trajectory.

Operational criteria for alignment categories were defined a priori to support systematic interpretation across data strands. Findings were categorized as convergent when quantitative results and qualitative themes indicated similar patterns, complementary when qualitative data expanded or explained quantitative findings, and divergent when results across strands differed. This integration approach enabled a comprehensive interpretation of feasibility and patient experiences within the dog assisted exercise therapy program.

As an exploratory trial, the study was not designed to detect small between-group differences but aimed to provide preliminary evidence on feasibility, acceptability, and potential outcomes of the intervention from both objective (questionnaire-based) and subjective (patient-reported) perspectives.

### Randomization

The random allocation sequence was generated by SW using an online randomization tool. Group assignments (intervention/control) were printed, placed in sealed envelopes, and stored at the hospital. After obtaining informed consent, a researcher (LJ) or clinical staff member opened the next numbered envelope to assign the patient to a group.

### Changes to trial protocol

Before trial commencement, the number of therapy sessions was reduced to six for feasibility. During the trial, the recruitment period was extended due to COVID-19 and staff changes. A qualitative component was added to complement the primarily quantitative design.

### Participants and setting

#### Participant recruitment

Participants were recruited between May 2021 and November 2024. We recruited the participants from a geriatric inpatient ward at an anthroposophical hospital specialized in integrative medicine.

Integrative medicine is a holistic approach to healthcare that combines conventional medical treatments with evidence-based complementary therapies to address the physical, emotional, mental, social, and spiritual aspects of health (National Center for Complementary and Integrative Health [34].

Anthroposophic medicine is a form of integrative medicine developed by Rudolf Steiner and Ita Wegman, which incorporates spiritual-scientific insights into diagnosis and treatment, aiming to support the patient's physical, psychological, and spiritual health alongside conventional medical care [21].

The dog was already integrated into the ward’s daily routine as part of the clinical setting before the study started.

#### In- and exclusion criteria

Following admission and completion of initial assessments, the attending physician assessed each patient for eligibility based on predefined inclusion and exclusion criteria. Inclusion criteria were: cognitive ability to provide informed consent, as determined by a standardized cognitive screening conducted as part of routine clinical assessment, sufficient proficiency in spoken and written German and the ability to complete the mobility and motility tests (i.e., independent mobility at room level). Exclusion criteria were: clinically relevant cognitive impairment preventing informed consent, diagnosed dementia, an aversion or prejudice against dogs or AAT, patients in isolation (due to COVID) and patients with known dog hair allergy. Patients who met the criteria were then invited to participate in the study and after giving their consent randomized to the intervention/control group.

#### Sample size

This study was designed as an exploratory randomized controlled mixed-methods trial with a planned sample size of 30 participants. The target was based on feasibility considerations and available resources rather than formal power calculations, as is common in exploratory or pilot trials.

Recruitment was stopped after enrolling 27 participants due to the scheduled end of the trial period. As a result, the final sample included 27 participants who were randomized and included in the analyses.

#### Setting and intervention

Patients in an acute geriatric inpatient ward received exercise therapy either with or without a Labrador therapy dog named “Willi.” The study was conducted in collaboration with Charité – Universitätsmedizin Berlin. The intervention group participated in six 30-min sessions of dog-assisted exercise therapy, while the active control group received six sessions of standard exercise therapy without the dog (see Table 1). The therapy dog was integrated into the sessions as part of the hospital’s geriatric complex treatment program.

**Table 1:** Content of each of the six exercise therapy sessions with and without the dog

Exercise Therapy with dog (intervention)	Exercise therapy without dog (active control)
Sound and sign signals for the dog	Occupational therapy (fine motor skills)
Mobilization of the legs	Mobilization of the legs
3 × Parcourse	3 × Parcourse
Walk	Walk

Both groups received an additional 16 sessions of the geriatric complex treatment alongside with integrative anthroposophic prozedures during their two-week geriatric rehabilitation program. The 16 sessions consisted of eight sessions each of physiotherapy and occupational therapy, along with neuropsychology, speech and language therapy, and anthroposophical therapies such as painting, eurythmy therapy, music therapy, footbaths, and wraps.

Exercise therapy, including AAT with the therapy dog Willi, involved bodily active sessions conducted in the presence of the dog during individual therapy. The dog was utilized in a supportive, motivating, and active role to enhance the therapy experience. Each intervention session was conducted exclusively by the trained therapy dog and its owner, a nurse, who together formed the therapy assistance dog team.

Feasibility was defined as the practical and procedural viability of implementing dog-assisted exercise therapy within routine geriatric inpatient care. This included recruitment capability, retention during the two-week program, session adherence, absence of serious adverse events, and integration into existing ward routines without major disruptions.

Feasibility was conceptualized as the practical implementability of AAT within routine geriatric rehabilitation (e.g., recruitment, retention, session delivery, safety), while acceptability referred to patients’ subjective evaluation of the intervention (motivation, satisfaction, perceived value).Due to the nature of the intervention (AAT), blinding of participants and care providers was not possible, as the presence of the therapy dog was clearly visible.

The dog’s owner, who served as the head nurse on the geriatric ward, also provided outreach nursing services in geriatrics. The dog and the nurse completed therapy dog training, which concluded with a test to assess their suitability as a therapy team in AAT. The dog was present on the ward most of the time when the nurse was on duty. Outside of the study, the dog was already integrated into the ward environment, moved freely, and was able to withdraw when needed. It had a blanket near the registration desk and a designated corner in the nurses’ room as a protected retreat area.

### Data collection and measures

The data collection process included two parts: first, the quantitative methods and measures, followed by the applied qualitative approaches.

#### Quantitative data collection

We exploratorily evaluated mobility, motivation, satisfaction and, if present, depression.

##### Mobility

Mobility was assessed using the Timed Up and Go (TUG) test, a validated tool for evaluating mobility and fall risk in geriatric populations [23]. As part of the geriatric assessment, it reflects muscle strength, joint function, and balance. Participants stood up from a chair, walked three meters, turned, returned, and sat down,walking aids were allowed, but no personal assistance. Time to complete the task was recorded. Interpretation followed standard cut-offs: < 10 s (no impairment), 10–19 s (mild), 20–29 s (clinically relevant), ≥ 30 s (severe). It was assessed at baseline and after the intervention.

##### Motivation and satisfaction

Motivation and satisfaction were assessed after each exercise therapy session using a visual analog scale (VAS; 0–100 mm), where 0 indicated "not motivated/satisfied" and 100 mm "very motivated/satisfied." Participants marked a point/cross on the line to reflect their response. The VAS has been widely used to assess subjective constructs that are continuous, experiential, and not easily captured using categorical response formats, including pain [9], anxiety [1], and motivation to eat [40]. VAS instruments are considered particularly suitable for such outcomes due to their sensitivity to small changes, ease of administration, and low respondent burden, all of which were important considerations in the context of the present study. VAS measures are frequently used in clinical and research settings with reliability and validity for disability assessment [8] and are critically discussed in terms of clinical relevance and evaluation [17].

##### Depression

Depression symptoms were assessed using the German version of the Hospital Anxiety and Depression Scale – Depression subscale (HADS-D) [18]. The HADS-D consists of 7 items rated on a 4-point Likert scale (0–3), with total scores ranging from 0 to 21. A score of 8 or above indicates possible depression, but a clinical psychological evaluation is required to confirm diagnosis. The HADS-D is a validated screening tool widely used in medical and research contexts. It was assessed at baseline and after the intervention.

#### Qualitative data collection and procedure

Qualitative data were collected at the end of the two-week inpatient stay using purposeful sampling to ensure representation from both study arms. In line with the mixed-methods design, qualitative interviews were conducted with participants from the overall study to complement the quantitative findings by exploring the feasibility and acceptability of the intervention, as well as participants’ experiences and perceptions of its therapeutic effects. Control group participants were included to assess the feasibility of exercise therapy with and without a therapy dog in the geriatric rehabilitation setting. Although the dog was not continuously present, its intermittent presence on the ward may have influenced participants’ overall experiences, including those in the control group, highlighting potential contextual effects beyond structured therapy sessions. Given the shared inpatient environment, some degree of contextual exposure could not be fully excluded. Including both groups therefore allowed us to explore potential contextual or contamination effects, as well as similarities and differences in the perception of exercise therapy between participants who received AAT and those who did not.

During the study briefing, patients were informed about the semi-structured interviews and encouraged to decide in advance if they wished to participate. At the end of the intervention, the study coordinator approached each patient to confirm their willingness to participate. A total of 10–15 interviews were planned to achieve thematic saturation. Interviews were conducted by trained researchers (M.Sc. in psychology and environmental psychology) and took place in a private setting chosen by the participant (room of the patients/a free room at ward/outside in private).

We conducted semi-structured interviews in person lasting 30,8 Minutes mean (min. 10, max. 54 min) per patient at the end of the inpatient stay.

#### Interview content and guide development

The semi-structured interview guide was developed based on existing literature, the study protocol, and expert consultations. It explored patients' emotional experiences during rehabilitation, including hospitalization reasons, notable memories, and interactions with the therapeutic team and the ward dog, Willi. Topics included initial contact, emotional responses, and perceptions of the dog’s presence. Participants were also asked about prior pet experiences and, for the intervention group, perceptions of AAT. Additional sections addressed mental health (e.g., mood, coping, loneliness), exercise therapy, and reflections on the rehabilitation experience. The interview guide is available in the Appendix 1.

### Data analysis

#### Quantitative data analysis

Demographic data (age, gender, diagnoses, residence) were collected at baseline. Data were transferred from paper forms to electronic tables, cleaned, and analyzed using IBM SPSS Statistics (Version 30.0.0.0). Due to the small sample size (*N* = 27), analyses were exploratory. Normality was assessed (e.g., Shapiro–Wilk test). Descriptive statistics, cross-tabulations, and group comparisons (unpaired *t*-tests or Mann–Whitney *U* tests) were conducted. Spearman’s ρ was used to assess the correlation between motivation and satisfaction. The evaluation is based on the intention-to-treat principle (ITT) [3, 22], without replacing missing values.

#### Qualitative data analysis

The interviews were audio-recorded, digitally stored in an encrypted format on the Charité – Universitätsmedizin Berlin servers, in compliance with data protection regulations, and after deleted from the recording device. Data was transcribed and pseudonymized externally and then analyzed with content analysis [25] using the MAXQDA® software program. All data were preserved and stored in accordance with institutional data protection policies and relevant legal regulations. After analysis, the recordings were deleted.

Procedural standardization was implemented to enhance transparency, coordination, and traceability of the research process. Ongoing discussions within the core research team and the interdisciplinary qualitative research working group of the Charité – Universitätsmedizin Berlin supported consistent decision-making across all phases of the study, including study design, development of the interview guide, data analysis and interpretation, and reporting. In addition, each interview was documented and reviewed using standardized qualitative interview protocols immediately after completion to ensure systematic documentation and procedural clarity.

At the same time, reflexivity constituted an epistemological commitment guiding the interpretive process. Team discussions were not aimed at achieving objectivity or consensus in a positivist sense, but at critically examining how researchers’ disciplinary backgrounds, assumptions, and positions shaped data generation and analysis. Thus, structured documentation supported transparency and coordination, while reflexive engagement ensured critical awareness of the interpretive nature of qualitative knowledge production.

Coding was conducted line by line by the primary analyst (LJ), who developed themes in an iterative process. Coding decisions and thematic structures were continuously discussed with a second researcher (EJ) and within the broader working group to enhance analytic depth and credibility. The appraisal process was not conducted independently by multiple coders but included peer debriefing and consensus-building.

### Data collection timing

Data were collected after randomization at baseline (T0) and after the intervention (T1). At T0, assessments included mobility, measured using the TUG test, and depressive symptoms, measured using the HADS-D. After each of the six intervention sessions (Tx), participant satisfaction and motivation were evaluated using a visual analogue scale (VAS). Follow-up assessments (T1) were conducted at the end of the inpatient stay, approximately two weeks after the start of the intervention. At T1, mobility (TUG) and depression (HADS-D) were reassessed to evaluate potential changes over the course of the intervention.

### Ethical considerations

During the intervention, the therapy dog interacted with the patients only in the presence of its owner. The owner carefully monitored the dog's signals and body language to identify and address any signs of stress or overexertion at an early stage. The owner followed the guidelines of the professional association “Therapiebegleithunde Deutschland e.V.” (TBD), a professional German association for therapy dogs that assist and support individuals in various therapeutic settings. Additionally, the therapy dog undergoes a health check every three months and an MRSA screening once a year. Methicillin-resistant Staphylococcus aureus (MRSA) is a type of bacteria resistant to many antibiotics, including methicillin, and can cause infections that are difficult to treat due to this resistance.

#### Ethics approval and consent to participate

The study was approved by the Charité – Universitätsmedizin Berlin ethics committee (EA4/071/21) on 22 April 2021, in line with national and institutional ethical standards and the Declaration of Helsinki. Participants provided written informed consent prior to participation. Data protection measures complied with the General Data Protection Regulation (GDPR) and institutional privacy policies. All procedures adhered to current clinical guidelines and Good Clinical Practice (GCP) standards. The study followed the COREQ checklist for reporting qualitative research to ensure methodological rigor and transparency [41, 42] and the CONSORT checklist for quantitative research [19].

#### Assessment of harms

Harms were defined as any physical, psychological, or behavioral adverse events potentially related to AAT, such as allergic reactions, fear, anxiety, aggression, injury (e.g., falls, scratches), or agitation.

In this study, harms were assessed non-systematically through informal reports by clinical staff, therapists, and animal handlers involved in the sessions, as well as through general observation during and after the intervention periods. There was no standardized adverse event reporting form or checklist used.

#### Safety and session adherence

Safety was assessed as a core feasibility outcome. Any adverse events or unintended effects occurring during the intervention period were non-systematically monitored and documented throughout all sessions. Intervention providers recorded all incidents in the overall therapy documentation, including the nature, timing, and severity of any events, as well as their potential relation to the intervention.

Session adherence was assessed by documenting participant attendance and engagement in each scheduled exercise therapy session. Attendance was recorded using standardized session logs completed by the treating therapists after each session. Adherence was calculated as the proportion of attended sessions relative to the total number of scheduled sessions for each participant. This approach allowed for a structured and consistent evaluation of intervention uptake across both study arms.

## Results

### Sample description: quantitative study

**Fig. 1:**
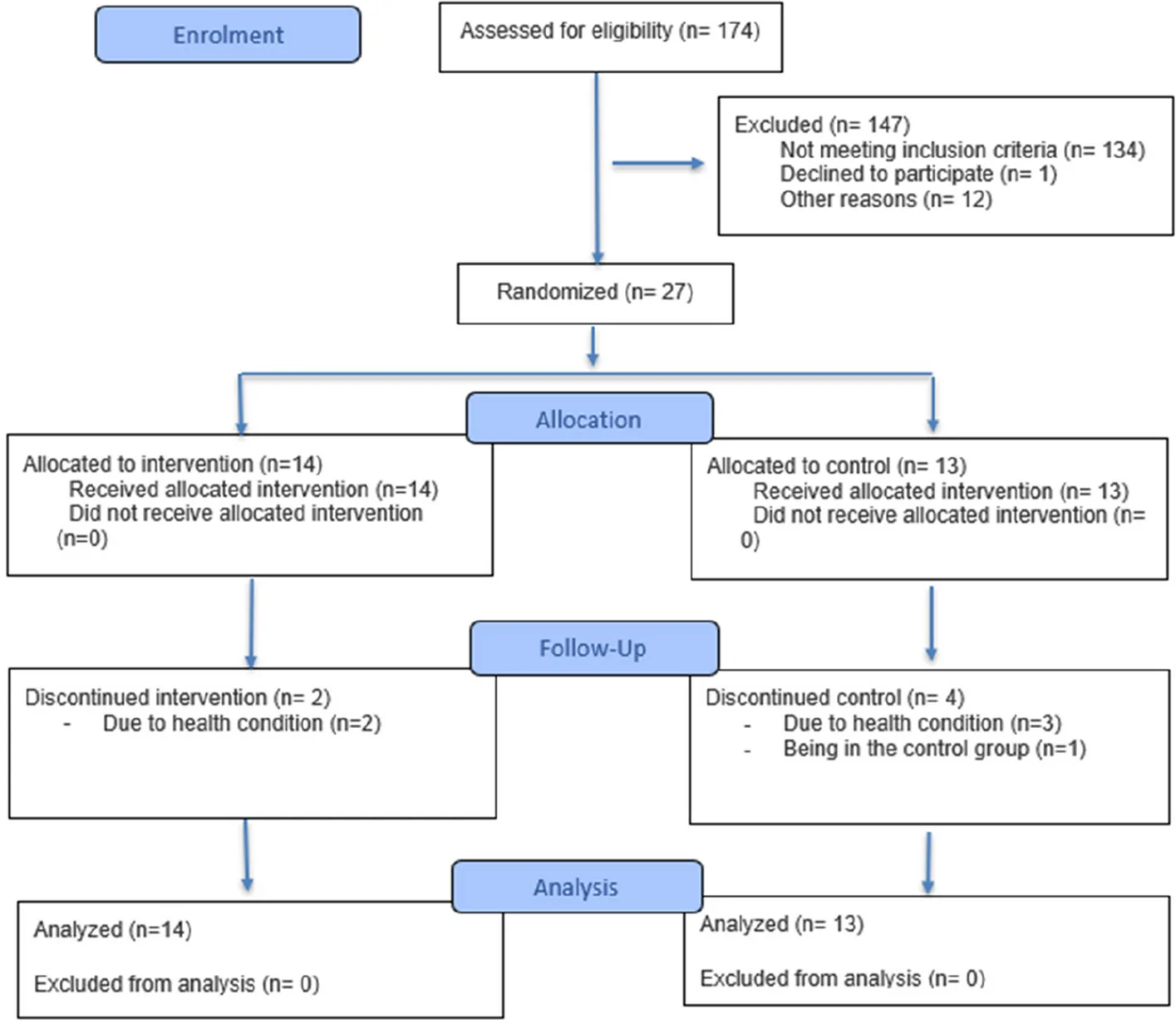
Participant CONSORT flow diagram from recruitment to analysis

A total of 27 patients participated, with 14 in the intervention group and 13 in the control group (see Fig. 1). Six participants dropped out due to health issues, five were transferred to other wards, and one declined participation after not being assigned to the intervention group.

The intervention group included 11 females and 3 males; the control group had 9 females and 4 males (See Table 2). The mean age was 83.93 years (SD = 7.04; range = 72–99) in the intervention group and 80.62 years (SD = 5.03; range = 72–88) in the control group. All participants met the predefined eligibility criteria, including sufficient cognitive ability to provide informed consent and participate in the intervention.

**Table 2:** Baseline data and participant characteristics

	**Intervention group (*n* = 14)**	**Control group (*n* = 13)**
Age, mean (SD)	83.9 (7.0)	80.6 (5.0)
Range	72–99	72—88
Sex: female male	11 3	9 4
Timed up and go (TUG), mean (SD)	18.3 (3.4)	20.8 (8.6)
HADS-D, mean (SD)	5.4 (4.1)	4.5 (3.7)

### Sample description: qualitative study

Eleven patients from the quantitative study participated in the qualitative interviews (M = 82 years; range = 75–93), including three male and eight female (see Table 3). Seven were in the intervention group (exercise therapy with the dog), and four in the control group (without the dog). The sample included a heterogeneous group of geriatric inpatients with conditions such as cancer, stroke, orthopedic issues, mental health disorders, COPD, accidents, neurodegenerative diseases, and chronic pain.

**Table 3:** Participant characteristics from the embedded qualitative study

	**N = **	**Intervention Group (*n* = 7)**	**Control Group (*n* = 4)**
**Gender:**			
Male	3	2	1
Female	8	5	3
Age (years)	11	Range 75–93 Mean 83.9	Range 75–88 Mean 81

### Mixed methods results

A mixed-methods analysis was conducted, in which quantitative and qualitative data were collected and analyzed separately before being integrated and interpreted through a joint display approach [13, 14]. This approach enabled a systematic integration of quantitative and qualitative findings across all participants. The results are organized into three main categories: (1) physical functioning, (2) psychological well-being, and (3) feasibility and acceptability, as outlined in Table 4.

**Table 4:** Joint display of quantitative and qualitative findings with explicit integration

Main Category	Quantitative Results	Qualitative Results	Integration
Physical Functioning	No significant between-group difference in mobility (TUG), *U* = 73.5, *p* =.81, *r* =.04	Participants reported increased engagement and perceived strength and awareness of limitations; dog encouraged movement	**Divergence/Complementarity:** No measurable short-term mobility changes, but motivational activation and increased strength and awareness of mobility limitations were evident
Psychological Well-Being	No significant difference in depression (HADS-D), *t*(15) = 0.12, *p* =.45, *r* =.03; baseline scores below clinical cutoff	Emotional comfort, security, and positive ward atmosphere attributed to dog; generally low loneliness	**Convergence (null effect):** No clinically relevant depressive symptoms. **Complementarity:** Emotional benefits perceived beyond standardized measures
Feasibility & Acceptability	High motivation (7–10) and satisfaction (8–10); no meaningful group differences (Δ = 0.1). Strong correlation between motivation and satisfaction (ρ =.65, *p* <.001)	High satisfaction with therapy; dog enhanced pleasure, engagement, and social atmosphere. Positive feedback across groups	**Convergence:** High quantitative motivation aligns with positive qualitative experiences. **Complementarity:** Dog strengthens therapeutic climate and engagement, supporting feasibility of AAT implementation

*AAT* Animal-Assisted Therapy, *TUG* Timed Up and Go Test, *HADS-D* Hospital Anxiety and Depression Scale–Depression subscale. Quantitative analyses included Mann–Whitney *U* tests, independent-samples *t* tests, and Spearman’s ρ correlations. Integration followed a convergent mixed-methods approach distinguishing convergence, divergence, and complementarity

## Physical functioning

### Quantitative results: mobility (TUG)

Mobility was assessed using the TUG test [23]. At baseline, median TUG scores were 17.00 s (IQR [15.50, 24.00])in the control group and 18.50 s (IQR [15.00, 21.25]) in the intervention group. Post-intervention, median TUG scores were 16.00 s (IQR [14.25, 22.50]) in the control group and 18.00 s (IQR [13.00, 20.50]) in the intervention group. Between-group comparison using the Mann–Whitney U test showed no statistically significant in change scores between groups (U = 73.5, Z = −0.25, *p* = 0.81, r = 0.04, see Appendix 2).

### Qualitative results: patients’ mobility perception

Participants perceived exercise therapy as beneficial, reporting increased strength, awareness of mobility limitations, and motivation to move. The presence of the therapy dog and support from the AAT therapist were highlighted as encouraging, enhancing engagement during and beyond therapy sessions. Participants noted comfort and enjoyment, even when exercises were challenging.One participant in the intervention group noted:

*“Yes, the (physical therapy sessions) were wonderful. I felt very comfortable. (I was) a bit unsure, which I found a bit strange — that I needed... help.”* (intervention group)

Similarly, a control group participant reflected on balance difficulties:

*“And I realized that (…) my balance was quite impaired. I also needed help. Normally, you don’t even notice that. But then I realized — wow — you need to work on that, you have to do something. Yes, that was difficult.”* (control group).

These reflections suggest that exercise therapy raised awareness of one’s own limitations.

The therapeutic function of the therapy dog served as a motivator for patients during AAT sessions and outside of structured interventions. Ward observations indicated that the dog positively influenced patient activity levels, with patients more engaged and willing to mobilize when the dog was present. As one participant noted:

*"I observed it with the older gentlemen or ladies, when Willi was there. Yes, they were mobilized, so to speak." (control group)*

These findings suggest that the presence of the therapy dog was perceived to encourage movement among geriatric patients, including outside formal therapy sessions, and may have contributed positively to the ward environment.

### Mixed-methods integration

Quantitative and qualitative findings provided both divergence and complementary perspectives within a feasibility framework. Divergence was observed in that quantitative mobility outcomes showed no between-group differences, whereas qualitative data indicated increased motivation, engagement in exercise, and greater awareness of physical capabilities and mobility limitations. At the same time, complementarity emerged, as qualitative findings helped contextualize how participants experienced the intervention within routine care and provided explanatory insight into motivational and experiential processes that were not captured by performance-based measures. Rather than indicating changes in physical performance, the integrated results suggest that animal-assisted exercise therapy may be relevant for understanding how patients engage with rehabilitation processes and perceive their therapeutic environment in an inpatient setting.

## Psychological well-being

### Quantitative results: depressive symptoms (HADS-D)

The HADS-D was evaluated using an unpaired t-test. There was no significant difference in depression scores between the intervention (M = 4.2, SE = 3.0) and control groups (M = 4.0, SE = 3.6), with a mean difference of 0.2 points (95% CI [–3.2, 3.6], SD = 3.05), which was not clinically relevant. The difference was not statistically significant (t(15) = 0.12, *p* = 0.45), and the effect size was extremely small (r = 0.03) (see Appendix 3). Scores were well below the clinical cutoff of 8 at baseline, indicating no clinically relevant depressive symptoms (see Appendix 2). Individual trajectories among the 11 interviewed patients showed variability: one patient improved markedly (15 → 4), one worsened (5 → 10), and one remained stable (0 → 0), reflecting heterogeneous responses.

### Qualitative results: dog's related emotional response

Participants reported emotional comfort and security associated with the therapy dog, highlighting perceived psychosocial benefits, even in the absence of measurable changes in depression scores.

Interviews revealed no notable differences between the groups regarding influence of the dog on patients’ loneliness. Most patients expressed comfort with solitude and did not perceive loneliness negatively, maintaining strong connections with friends and family.

Several participants emphasized the emotional value of the therapy dog’s presence. One participant expressed appreciation for the dog’s role:

*“Yes, the (dog) gave me security.”* (intervention group)

These reflections suggest that the therapy dog was a positive aspect of the rehabilitation experience.

### Mixed-methods integration

Convergence (null effect) was observed as both groups demonstrated no clinically relevant depressive symptoms. At the same time, complementarity emerged, as qualitative findings revealed perceived emotional benefits that were not captured by standardized depression scores. Together, these results suggest that AAT may enhance subjective well-being without producing measurable quantitative changes in depressive symptoms in a population that has no diagnosed depression.

## Feasibility and acceptability

### Safety, harms, session adherence, and retention

No serious adverse events related to the intervention were observed during the study period. Harms were defined as any physical, psychological, or behavioral adverse events potentially related to AAT, including allergic reactions, fear, anxiety, aggression, injury (e.g., falls or scratches), or agitation. Potential harms were monitored in a non-systematic manner through informal reports from the therapist and animal handler involved in the sessions, as well as through general observations during and after the intervention sessions. No relevant adverse events were reported in relation to the intervention.

Participant retention was high, with 21 of 27 participants (77,78%) completing the post-intervention assessment. Reasons for dropout included worsening health status and transfer to another ward (*n* = 5), as well as one participant withdrawing after being allocated to the control group.

Session adherence was overall high. Attendance at scheduled exercise therapy sessions was documented by treating therapists using standardized session logs. Adherence was defined as the proportion of attended sessions relative to all scheduled sessions per participant. In some cases, participation was not possible due to organizational restrictions related to COVID-19, including temporary closure of the ward, which led to missed sessions independent of participant willingness or intervention acceptability. In addition, due to the complexity of inpatient ward routines and the concurrent delivery of multiple (up to 16) other therapy interventions, there were occasional difficulties in coordinating fixed time slots and room availability for the intervention sessions. Despite these logistical constraints, overall attendance remained high, indicating good feasibility of intervention delivery within the inpatient setting.

### Quantitative results: motivation and satisfaction of exercise therapy with/without dog

Motivation and satisfaction were assessed across all six sessions. The mean between-group differences were 0.1 for motivation and 0.1 for satisfaction (both VAS), which are not clinically meaningful (see Appendix 2). Overall, participants reported high motivation and satisfaction levels throughout treatment, with only occasional lower ratings, suggesting generally stable acceptance of exercise therapy independent of condition.

A Spearman’s ρ correlation between motivation and satisfaction showed a strong positive correlation (ρ = 0.65, *p* < 0.001), indicating a significant association.

### Qualitative results: patient experiences with exercise therapy and therapy dog presence

Qualitative data supported the quantitative results by highlighting high perceived acceptability of exercise therapy and positive emotional responses to participation. Exercise sessions were commonly described as pleasant, although perceived intensity varied between individuals, ranging from “too easy” to “physically demanding”.

Participants consistently emphasized the positive emotional influence of the therapy dog on motivation and ward atmosphere. The dog’s presence was described as pleasurable, mood-enhancing, and socially activating, contributing to smiling, interpersonal interaction, and a more positive therapeutic environment—even without direct interaction. One participant explained:

*"Everything that gives pleasure contributes to it (motivation) and Willi gave pleasure. But as I noticed, it wasn't just me, the others too. (...) But I saw that (...) when Willi came around, the (people) all started smiling. (...) We didn't play with him, we didn't give him any treats, we didn't do anything, but he came to look, to check.* (intervention group)

Many participants also expressed a desire for more therapy dogs in hospitals. One control group patient stated:

*"It’s good (that the dog is on the ward). (…) There should be many more dogs here."* (control group)

Patient feedback on the dog was consistently positive. Both groups valued the emotional support and positive atmosphere created by the therapy dog. One participant reflected:

*“I notice how the animal brings a smile to people who are so closed off, who completely withdraw inwardly because of all the suffering they have experienced.”* (control group)

Patients described AAT as a positive component of their rehabilitation experience.. Participants in both groups were asked about the therapy dog Willi and whether they had any direct interaction with him. Willi was regularly present on the ward before the study started when his owner, a nurse, was on duty, but also had space to retreat when needed, in accordance with ethical guidelines. Although some control group participants had no or limited contact with the dog, they still shared detailed and emotional reflections about his presence. Many were initially surprised by the unexpected encounter with a therapy dog but responded positively, expressing joy and engaging in conversations about the dog.

### Mixed-methods integration

Integration of quantitative and qualitative findings demonstrated convergence and complementarity between data sources. Convergence was reflected in consistently high quantitative motivation and satisfaction scores, which aligned with qualitatively reported positive therapy experiences and overall acceptance of exercise therapy. Complementarity emerged as qualitative findings indicated that the therapy dog primarily enhanced the therapeutic climate, emotional engagement, and social atmosphere, thereby supporting the practical feasibility and acceptability of implementing AAT within clinical exercise settings. Quantitative feasibility outcomes further supported these findings, demonstrating high session adherence and participant retention (77.8%), despite organizational challenges related to COVID-19 restrictions and the complexity of inpatient scheduling. In addition, no intervention-related adverse events were observed, indicating that AAT could be implemented safely within this setting. Together, the integrated results suggest that AAT contributes mainly to experiential and contextual benefits while also demonstrating good feasibility and safety for implementation in inpatient exercise therapy, rather than producing measurable differences in motivational ratings.

## Discussion

This study aimed to evaluate the fesibility of exercise therapy with and without the inclusion of a therapy dog in geriatric inpatients using a mixed-methods design. Overall, findings support the feasibility of implementing animal-assisted exercise therapy in geriatric inpatient rehabilitation. Although no clinically meaningful quantitative improvements in mobility or depressive symptoms were observed, qualitative results indicated high patient engagement, positive emotional responses, and perceived experiential benefits associated with therapy participation. Together, these findings suggest that animal-assisted exercise therapy is feasible and well accepted in this population, with potential value primarily reflected in patient experience rather than measurable short-term clinical changes.

### Physical functioning

Quantitative and qualitative findings provided complementary perspectives on mobility-related outcomes. The quantitative results showed no between-group differences in TUG performance, which is expected given that the study was not designed or powered to detect intervention effects on mobility outcomes. Rather than indicating absence of change, these findings primarily reflect the methodological constraints of feasibility research. In contrast, qualitative findings provided complementary insights, suggesting that participants experienced increased engagement in physical activity during sessions, greater perceived physical capability, and enhanced awareness of their own mobility limitations. Participants also described that the presence of the therapy dog encouraged movement and contributed to a more active and motivating therapeutic environment. Taken together, the mixed-methods findings indicate that while no measurable short-term differences in mobility were observed, AAT may play a role in shaping patients’ subjective experience of movement and engagement with exercise therapy. These insights are valuable for informing the selection and operationalization of outcomes in future adequately powered trials.

In geriatric inpatient populations, mobility outcomes such as the Timed Up and Go (TUG) may also be influenced by natural functional fluctuations and underlying health status, which complicates interpretation of short-term changes in feasibility studies. Previous work has suggested minimal clinically important differences (MCIDs) in more homogeneous outpatient populations, such as individuals with hip osteoarthritis or chronic obstructive pulmonary disease (e.g., [30, 47]. However, the applicability of such thresholds to acutely or subacutely hospitalized older adults remains uncertain. Accordingly, future trials should carefully consider the selection and interpretation of mobility endpoints in this population, potentially combining performance-based measures with patient-reported and experiential outcomes.

Mobility is commonly a key focus in geriatric rehabilitation settings, and previous research has highlighted the potential value of interventions aimed at maintaining or improving functional capacity [32, 36]. In the present feasibility study, qualitative findings provided complementary insights into participants’ experiences of movement and exercise participation. Participants described increased engagement in physical activity during sessions, enhanced awareness of their own physical limitations, and a greater sense of motivation and emotional encouragement to participate in exercise. These experiences suggest that AAT may contribute to rehabilitation processes primarily through psychological and motivational pathways that shape how patients engage with therapy, rather than through directly measurable short-term changes in mobility performance. Notably, participants also reported perceived benefits even in sessions without direct interaction with the dog, indicating that the broader therapeutic atmosphere may have played an important role in shaping engagement and perceived well-being. These findings can inform the design of future studies by highlighting relevant experiential mechanisms and outcome domains for further investigation.

Patients in both groups reported emotional benefits associated with with the presence of the dog. However, the extent, nature and frequency of contact were not systematically recorded, and some patients reported no direct physical interaction. It therefore remains unclear whether reported experiences were related to direct contact or to the broader awareness of the therapy dog being present in the care environment. These qualitative observations suggest that contextual and environmental factors may have shaped patients’ experiences of the intervention and the ward atmosphere.

In line with the feasibility focus of this study, quantitative outcomes were not interpreted as indicators of intervention effects. Instead, mixed-methods integration was used to compare and contextualize feasibility-relevant findings across data sources. No intervention-related adverse events were observed, indicating that animal-assisted exercise therapy could be implemented safely in this inpatient setting. Feasibility outcomes further demonstrated high session adherence, despite organizational constraints related to inpatient care routines and intermittent COVID-19-related disruptions. Participant retention was also high, supporting the practicality of conducting this type of intervention in a clinical ward environment. Qualitative data indicated increased engagement in exercise activities, heightened awareness of physical capabilities and limitations, and greater perceived motivation to participate in therapy. Together, these findings suggest that animal-assisted exercise therapy may be acceptable to patients and feasible to implement in an inpatient setting, with its primary contribution lying in shaping engagement with rehabilitation processes and the therapeutic environment rather than in demonstrating short-term changes in objective physical performance outcomes.

### Psychological well-being

Mixed-methods findings suggest that animal-assisted exercise therapy is feasible and well accepted with regard to psychological well-being in geriatric inpatients. Mixed-methods integration revealed complementarity between data strands: while standardized depression measures showed neither a depression nor a measurable change, qualitative experiences pointed to emotional benefits and enhanced therapeutic engagement. This may be explained by the absence of clinically diagnosed depression within the sample and the limited duration and intensity of therapy during acute hospitalization, which likely restricted measurable psychological change. Nevertheless, the systematic review and meta-analysis by Villarreal-Zegarra et al. [43] reported reduction in depressive symptoms following AAT.

In contrast, qualitative findings indicated perceived emotional and social benefits associated with therapy participation and the presence of the therapy dog. Participants described improved mood, emotional comfort, and increased openness during rehabilitation, suggesting that AAT may positively influence psychological well-being through supportive and relational mechanisms rather than short-term symptom reduction. The therapy dog appeared to facilitate engagement particularly among patients less receptive to conventional therapeutic approaches, highlighting its potential as an accessible motivational component within rehabilitation settings.

### Feasibility and acceptability

Mixed-methods findings indicate high acceptability and feasibility of exercise therapy in geriatric inpatients, independent of therapy condition. Quantitative results demonstrated consistently high motivation and satisfaction across sessions, suggesting that structured exercise therapy was generally well received. Lower engagement appeared to be associated with broader psychosocial factors, such as loneliness, highlighting that individual emotional burdens may influence therapy participation beyond the intervention itself. Loneliness, particularly in geriatric patients, can affect emotional well-being, leading to feelings of isolation and disconnection, which may undermine motivation for therapy [26, 31, 38]. This suggests that while AAT can have a positive influence on overall well-being, it may not fully address the underlying emotional challenges such as loneliness, which could require more targeted interventions to improve engagement and satisfaction in exercise therapy.

Qualitative data complemented these findings by emphasizing the positive perception of the therapy dog across both groups. Participants described the dog’s presence as mood-enhancing and socially activating, contributing to a more supportive ward atmosphere and facilitating interpersonal interaction. These experiences suggest that AAT may strengthen motivation indirectly by improving the emotional and social rehabilitation context rather than altering motivation ratings alone. Reports that the therapy dog influenced mood and engagement beyond structured sessions further indicate potential environmental effects within routine clinical care. Rodrigo-Claverol et al. [36] and Ambrosi et al. [4], demonstrated that animal presence encourages communication and fosters positive emotional responses. Moreover, their findings indicated that AAT could positively influence patients' perceptions of their illness tragectory and sense of control over treatment, potentially enhancing feelings of empowerment. Our results support this, indicating that the therapy dog possibly enhanced interactions among patients, promoting a sense of connection and contributing to a more supportive hospital environment. However, it should be noted that examining these broader environmental and social effects was not the primary objective of the qualitative component.

In addition to acceptability findings, feasibility outcomes further support the safe and practicable implementation of the intervention in an inpatient geriatric setting. No serious adverse events related to the intervention were observed, and no relevant intervention-related harms were reported. Participant retention was high (21/27, 77.8%), with dropouts primarily due to clinical deterioration and ward transfer. Session adherence was also consistently high, despite organizational constraints related to inpatient routines and intermittent COVID-19-related disruptions. Together, these findings indicate that the intervention can be delivered safely with good participant retention and strong adherence under real-world clinical conditions, supporting its feasibility in routine inpatient rehabilitation contexts.

Identifying the active mechanisms underlying AAT is critical for establishing its efficacy and informing clinical application. Wagner, Grob, and Hediger [45] highlighted the importance of differentiating between specific factors—such as the presence of the animal—and non-specific factors—such as the therapeutic context and relationship. They questioned whether observed benefits are attributable primarily to the animal itself or to broader aspects of the therapeutic environment. Consistent with this perspective, our findings suggest that non-specific factors such as (1) general therapeutic aspects, (2) social interaction, (3) physical activity, and (4) novelty, likely played an important role in influencing outcomes. López-Cepero [28] similarly underscored the need to examine underlying mechanisms in AAT. By employing an active control group, our study aimed to isolate and examine the specific contribution of human–animal interaction within the multifaceted context of AAT.

### Animal-assisted place attachment

The presence of a therapy dog can act as a catalyst for social engagement, relaxation, safety, and joy, contributing to overall well-being. A relevant theoretical framework that may help explain these effects is place attachment, defined as the emotional bond between individuals and specific environments [39]. Emotional connections to a ward or a therapeutic setting may develop through repeated, and meaningful interactions, such as those involving a therapy dog. These connections can support patients’ comfort and engagement in clinical environments.

This theory supports our findings, as patients reported that the presence of the dog was beneficial—regardless of how frequently the dog was present on the ward or whether patients belonged to the intervention or control group. The mere presence of the dog appeared to promote a positive emotional connection to the environment, aligning with the concept of place attachment. Furthermore, studies support the idea that place attachment plays a crucial role in the rehabilitation process [33]. Similarly, Rohani & Shaliamini [37] demonstrated that integrating principles of place attachment into modern hospital design can lead to faster patient recovery, greater job satisfaction among hospital staff, improved medical education, and enhanced environmental quality within the hospital setting. The therapeutic presence of animals, such as therapy dogs, may support the development of such attachments within clinical environments.

In summary, the presence of a therapy dog may strengthen place attachment and emotional safety, fostering motivation, resilience, and engagement in therapy. Human–animal interaction is not merely an adjunct to treatment but a meaningful component of how patients experience and respond to their therapeutic environment.

### Novelty effect as a potential influence

The presence of a therapy dog in a hospital may introduce a novelty effect, potentially enhancing patient motivation simply due to its unexpected or uncommon nature. This effect could have influenced participants' responses in our study, as AAT remains relatively rare in clinical inpatient settings. Previous research suggests that AAT is particularly susceptible to novelty-driven responses, which may threaten construct validity [29]. Contextual and psychosocial factors—such as patient expectations, prior knowledge, and treatment setting—can significantly shape outcomes [44, 46]. These influences may lead to placebo-like effects, where perceived benefits are driven more by the experience or presentation of the intervention than its active components. Understanding the potential impact of novelty is essential when evaluating AAT.

### Pet ownership as an influencing factor

While AAT involves structured, goal-oriented interventions delivered by professionals in clinical settings, pet ownership (PO) represents an informal, ongoing relationship with animals. Both AAT and PO can influence well-being and physical activity, though their mechanisms and contexts differ substantially. AAT is typically implemented to achieve specific therapeutic outcomes, whereas PO provides continuous companionship and emotional support without structured therapeutic input.

In our qualitative group, 9 of 11 participants were previous pet owners (not limited to dogs), which may have influenced their perceptions and responses to the intervention. Prior experiences with animals could have shaped their receptiveness to the presence of the therapy dog, regardless of group allocation. Moreover, research suggest that pets play a multifaceted role in mental health. Hawkins et al. [16] identified various positive effects of pet ownership, including increased motivation and behavioral activation, enhanced social connectedness, reduced loneliness, and support in coping and recovery. However, they also emphasize that pets are not a substitute for mental health treatment and may have varying impacts depending on individual circumstances. Mental health professionals are thus encouraged to recognize the nuanced role of animals in individuals’ lives.

The contrast between AAT and PO highlights the importance of context and individual history in understanding the therapeutic potential of human–animal interactions.

### Strengths and limitations

A key strength of this study is its mixed-methods design, which enabled the systematic integration of quantitative and qualitative findings and provided insights into the feasibility and acceptability of the intervention that would not have been captured by quantitative measures alone. While no measurable short-term changes were observed in mobility or depressive symptoms, the qualitative findings demonstrated high participant acceptability and offered a deeper understanding of patients' experiences with the intervention. Participants consistently described the therapy dog as a motivating and valued component of exercise therapy and reported positive emotional and social experiences. These findings complement the quantitative results and generate hypotheses for future studies to investigate whether these perceived benefits are associated with measurable clinical outcomes.

However, several limitations must be acknowledged. Randomization post-consent led to one dropout when the participant was assigned to the control group. A true control condition could not be fully established, because the therapy dog was occasionally visible patients on the ward (0–3 times/week), and participants in the control group were therefore aware of its presence, potentially introducing contamination effects. Our qualitative findings indicate that this contextual awareness may have influenced participants’ perceptions and experiences to some extent. In addition, informal exposure to the dog may have influenced mood and engagement, confounding between-group comparisons. The extent of these interactions was not systematically assessed, limiting our ability to quantify their potential impact.

A potential sampling bias may have occurred, as most participants were pet owners, possibly increasing receptivity to the intervention. While two participants had previous negative experiences with dogs, all expressed positive views toward the therapy dog Willi.

COVID-19-related disruptions (e.g., isolation, staff shortages) impacted session delivery and continuity. Especially the occasional unavailability of the AAT team due to illness of either the therapist or the therapy dog, which led to interruptions in the intervention schedule. One patient received only two sessions due to quarantine restrictions but still reported a positive perception.

The lack of significant change in depression scores may reflect low baseline symptom severity. Additionally, missing data (e.g. drop outs) and a small sample size limit statistical power and generalizability. Larger trials are needed to identify subgroups most likely to benefit from AAT and to examine its potential for enhancing treatment motivation, place attachment, and engagement in geriatric populations.

### Implications for practice and further research

Implementing exercise therapy with a therapy dog in clinical settings presents logistical challenges, including coordinating staff schedules, therapy spaces, and ensuring hygiene and safety protocols. Ethical considerations – such as appropriate rest periods for therapy animals and clear withdrawal protocols—must also be addressed (see [45]. Additionally, intake procedures should include screening for allergies, phobias, and past experiences with animals to ensure appropriate and individualized care [27, 48].

Future studies should include larger sample sizes, informed by formal power calculations, to enable detection of meaningful effects. Employing video observation and multi-perspective interviews (patients, staff, physicians) would allow a more comprehensive assessment of how therapy dogs influence mobilization, social interaction, and the overall ward atmosphere. Tracking patient–dog interaction time, both within and outside structured sessions, would clarify the mechanisms behind AAT effects. Contextual factors—such as prior pet ownership, staff engagement, ward culture, and novelty—should also be accounted for.

Longitudinal designs with repeated interviews could explore how AAT supports place attachment and emotional safety over time, particularly across facilities with different levels of animal integration (e.g., visiting vs. resident animals). Observing patient–patient interaction may further reveal the animal’s role in fostering social bonds and a more cohesive ward environment.

## Conclusion

This mixed-methods study demonstrates the feasibility and high acceptability of animal-assisted exercise therapy in geriatric inpatient rehabilitation.The integrated findings indicate that the intervention was well received by participants, with patient-reported benefits primarily reflecting experiential and psychosocial aspects.Quantitative findings showed consistently high levels of motivation and satisfaction across both conditions, while no measurable short-term changes in mobility or depressive symptoms were observed.

In contrast, qualitative results revealed psychosocial and behavioral benefits from patients’ perspective, including enhanced motivation, emotional well-being, social interaction, and engagement in therapy. Mixed-methods integration highlighted patterns of convergence regarding the high acceptability of the intervention and complementarity between the quantitative and qualitative findings. Although no measurable short-term changes were observed in the quantitative outcomes, participants consistently perceived the therapy dog as contributing positively to the therapeutic environment, patient activation, and perceived functional awareness.

These findings suggest that AAT may support rehabilitation through contextual and relational mechanisms, fostering a positive ward atmosphere and encouraging participation. Participants consistently perceived the therapy dog as contributing positively to the therapeutic environment and to their rehabilitation experience. These observations suggest potential contextual and relational mechanisms that warrant further investigation rather than demonstrating clinical benefit. The findings generated by this feasibility study provide hypotheses for future adequately powered trials to examine whether these perceived benefits are associated with improved engagement during rehabilitation and whether they translate into measurable clinical outcomes over longer intervention periods. Further research with larger samples and longer intervention periods is needed to clarify underlying mechanisms and to determine its impact on long-term clinical outcomes.

## Data Availability

The individual de-identified participant data (including the data dictionary), statistical codes, and any other related materials are securely stored in accordance with applicable data protection regulations. Quantitative and qualitative data are digitally stored in an encrypted format on the servers of Charité – Universitätsmedizin Berlin. The key linking participant identities to the de-identified data is maintained separately in a secure, locked storage at the hospital and the affiliated research institute. The data and material generated and analysed during this study are partially included in this published article and its supplementary information files in pseudonymised form. Additional data are available from the corresponding author (LJ) or the PI (HM) on reasonable request, subject to institutional and ethical regulations.

## Appendix 1

### Interview guide

Getting started

- Feelings during the rehabilitation period
- Reason for hospitalization
- Special memories

Therapeutic team/dog

- Contact with the ward dog Willi
- Getting to know Willi (situation, feelings)
- Opinion on the dog's presence
- Own experiences with pets
- For intervention group: Perception of dog therapy
- Positive/negative aspects

Mental health

- Mood before the stay
- Mood during the stay
- Dealing with changes
- Topic of loneliness

Exercise therapy

- Opinion on the exercise therapies
- Feelings during/after the therapies
- Particularly positive/negative situations

Conclusion

- Insights and benefits of the stay

## Appendix 2

### Table Post-Intervention Scores

**Table 5:** Post-Intervention Scores for Mobility and Depression, and Mean Motivation and Satisfaction Scores Across Sessions 1–6 in the Intervention and Control Groups

	**Intervention group** **(** ***n*** ** = 14)**	**Control group** **(** ***n*** ** = 13)**	**Difference between groups**	***p*** ** value**
	Mean (SD) Post-Test	Mean (SD) Post-Test		
TUG (Mobility)	17.2 (3.4)	18.8 (7.6)	-1.6	*p* = 0.81
HADS-D (raw value)	4.2 (3.0)	4 (3.6)	0.2	*p* = 0.45
VAS Motivation 1–6	9.0 (0.6)	8.9 (1.3)	0.1	
• VAS Motivation 1	8.6 (0.9)	9.3 (0.9)	-0.7	*p* = 0.5
• VAS Motivation 2	9.1 (0.5)	9.0 (0.6)	0.1	*p* = 0.7
• VAS Motivation 3	9.1 (0.4)	8.9 (1.0)	0.2	*p* = 0.2
• VAS Motivation 4	9.1 (0.5)	8.8 (1.4)	0.3	*p* = 0.6
• VAS Motivation 5	9.0 (0.3)	8.4 (2.3)	0.7	*p* = 0.5
• VAS Motivation 6	9.0 (0.8)	9.0 (1.2)	0	*p* = 0.7
VAS Satisfaction 1–6	9.0 (0.7)	8.9 (1.2)	0.1	
• VAS Satisfaction 1	9.0 (0.7)	9.2 (1.0)	-0.2	*p* = 0.9
• VAS Satisfaction 2	9.0 (0.7)	8.6 (1.4)	0.4	*p* = 0.8
• VAS Satisfaction 3	8.4 (1.7)	8.9 (1.0)	-0.5	*p* = 0.4
• VAS Satisfaction 4	9.0 (0.5)	8.9 (1.3)	0.1	*p* = 0.6
• VAS Satisfaction 5	9.0 (0.4)	9.1 (1.0)	-0.1	*p* = 0.9
• VAS Satisfaction 6	9.3 (0.3)	8.9 (1.4)	0.4	*p* = 0.8
